# Warm Temperature Increments Strengthen the Crosstalk between Roots and Soil in the Rhizosphere of Soybean Seedlings

**DOI:** 10.3390/plants12244135

**Published:** 2023-12-12

**Authors:** Wanying Zhao, Mingxing Wen, Caitong Zhao, Shurui Zhang, Runa Dou, Xuefeng Liang, Xianfeng Zhang, Zhihua Liu, Zhenfeng Jiang

**Affiliations:** 1 Key Laboratory of Soybean Biology in Chinese Ministry of Education, Key Laboratory of Soybean Biology and Breeding/Genetics of Chinese Agriculture Ministry, College of Agriculture, Northeast Agricultural University, Harbin 150030, China; S220301053@126.com (W.Z.); WenMingXing0302@163.com (M.W.); 18846773398@163.com (C.Z.); 15765363534@163.com (S.Z.); douruna@163.com (R.D.); liangxf1230@163.com (X.L.); 2 The Training Center of the Undergraduate, Northeast Agricultural University, Harbin 150030, China; 0451zhangxianfeng@163.com; 3 College of Resources and Environment, Northeast Agricultural University, Harbin 150030, China

**Keywords:** soybean, roots, in situ enzyme spectrum, soil microorganisms, rhizosphere enzyme activity

## Abstract

The plant rhizosphere underlies the crosstalk between plant and soil and has a crucial role in plant growth and development under various environments. We examined the effect of temperature rise on the rhizosphere environment of soybean roots to clarify the rhizosphere crosstalk between roots and soil in response to warm temperature rises in a global warming background. The in situ results of root enzyme activity revealed that soybean roots secrete β-glucosidase, and enzyme spectrum imaging demonstrated different enzymatic activities under different temperature environments. The soil enzyme kinetics results showed that soil enzymatic activity increased with increasing temperature, and soybean rhizosphere soil enzymatic activity was higher than that of non-rhizosphere soil. Rhizosphere soil and non-rhizosphere soil showed that the dominant bacterial phylum in soybean rhizosphere soil was *Acidobacteria*, and the dominant bacterial genus was JG30-KF-AS9. Compared with non-rhizosphere soil, rhizosphere soil was more nutrient-rich, and root secretions provided abundant carbon sources and other nutrients for soil microorganisms in the rhizosphere. Rhizosphere microorganisms affect plant growth by influencing the decomposition of soil organic carbon. The organic carbon content of rhizosphere soil was higher than that of non-rhizosphere soil under high temperatures.

## 1. Introduction

The root system plays a key role in the environmental adaptation of crops by promoting the effective absorption of soil nutrients and water [1,2,3]. Plant roots also secrete metabolites into the rhizosphere [4], which satisfy the activity of microorganisms and enzymes [5], resulting in enzyme hotspots [6,7]. The intracellular enzymes are released to the rhizosphere; thus, crosstalk with the roots and microbial organisms occurs in the enzyme hotspot [8]. Plant–microbe interactions play an extremely important role in soil fertility, health, and plant growth and development [9]. Root exudates are not only intermediaries in the interaction between plants and rhizosphere microorganisms but also constitute the root system of plants. As the main factors affecting the activity of rhizosphere microorganisms, root exudates often serve as a carbon source and energy source for the growth and reproduction of rhizosphere microorganisms, and affect the type, quantity, and distribution of microorganisms in the plant rhizosphere. Moreover, as a high-energy habitat, the rhizosphere, where 20% of plant photosynthesis products are secreted from the roots, harbors a higher concentration of microorganisms than the bulk soil [10,11]. Microbes produce a variety of signaling molecules to affect the development of plant roots and alter their architecture [12]. These chemical compounds act as messengers, allowing plants to talk with microbes and tailor the microbiome within and around the roots to meet plants’ specific needs.

Soil enzymes are the primary biological driving force for organic matter decomposition and nutrient cycling [13], and their activities reflect the intensity and direction of various biochemical reactions [14]. Therefore, enzymatic activity is used as a sensitive indicator reflecting soil fertility and ecosystem function. Soil enzymes in the rhizosphere originating from microbial cells and plant roots have certain catalytic functions to mobilize soil nutrient stores [15]. Although the activity of rhizosphere soil enzymes is high, it remains unclear whether the mechanisms by which microorganisms break down organic matter differ in the hotspots and soils. This was revealed by enzymatic kinetic parameters [16,17], which described catalytic activity and enzyme–substrate affinity [18,19], as well as the possible distribution of rhizosphere enzymatic activity [20,21,22]. Higher soil enzyme activity indicates strong carbon transformation in the soil, thereby promoting plant growth [23]. The mineralization of soil organic carbon is driven by soil microorganisms and mediated by soil enzymes to increase soil carbon storage and organic matter stability [24]. Soybeans are considered a rich source of isoflavones. β-glucosidase (β-d-glucoside glucohydrolases; EC 3.2.1.21) is an enzyme that catalyzes the hydrolysis of the β-glycosidic linkage from the non-reducing end of isoflavone glucosides. Therefore, soil enzyme activity, such as that of β-glucosidase (EC 3.2.1.21), is significantly positively correlated with soil organic carbon content [25] and is used to monitor soil quality [26,27,28,29].

Temperature rises resulting from global climate change seriously affect crop yields and threatens the food supply [30]. Temperature changes altered the composition and functional structure of soil microbial communities [31] and reduced the abundance and diversity of fungi and actinomycetes in the soil [32]. Several studies have shown that temperature rises indirectly affect soil enzymatic activity by changing the composition and structure of soil microbial communities [33]. The kinetic constant of cellulose was inhibited at low temperatures, leading to a direct reduction in soil enzymatic activity [34]. It has been generally believed that increasing temperature may improve soil enzymatic activity [35], which may have resulted from the response of soil enzyme activity to temperature increase and may also be constrained by other factors. However, there has been little research progress regarding factors related to the growth of soybean roots, and further in-depth studies are urgently needed. Therefore, in this study, we used in situ enzyme activity to analyze the activity of β-glucosidase in the taproot and lateral root of soybeans at different temperature environments with the aim of reducing fertilizer application, promoting crop growth and development, and guaranteeing high and stable yields for soybeans.

## 2. Results

### 2.1. In Situ Determination of Root Enzyme Activity

#### 2.1.1. Determination of the β-Glucosidase Source

**Figure 1:**
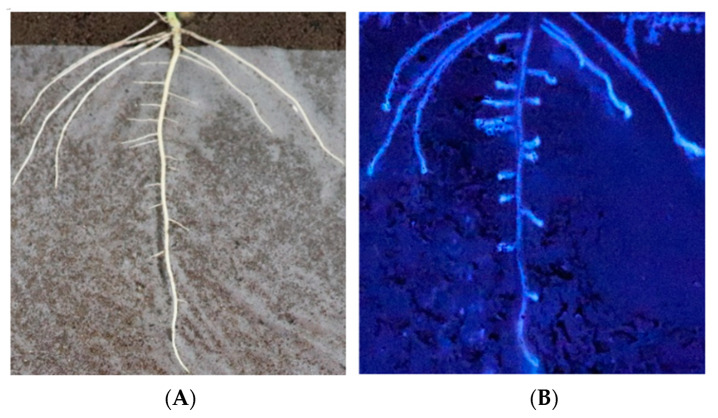
Evidence that soybean roots secrete β-glucosidase into the rhizosphere. (**A**) The root architecture on the film. (**B**) β-glucosidase activity along the root.

In situ analysis of enzymatic activity could accurately reflect enzyme changes, so in situ analysis of β-glucosidase activity was performed. The in situ results of enzyme activity carried out by nylon membrane isolation of the soil and soybean roots showed that the soybean roots could secrete β-glucosidase to the root surface (Figure 1), participate in the changes in microbial species and abundance in rhizosphere soil, affect the types and abundance of rhizosphere nutrients, and feedback plant growth and development.

#### 2.1.2. Enzyme Activity Increased along the Taproot from the Root Base to the Root Tip

**Figure 2:**
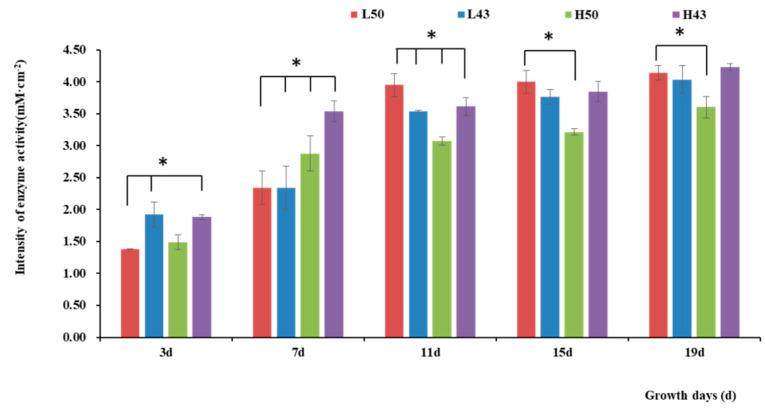
Intensity of β-glucosidase activities in the taproot base. * indicates significant at the 0.01 probability level.

**Figure 3:**
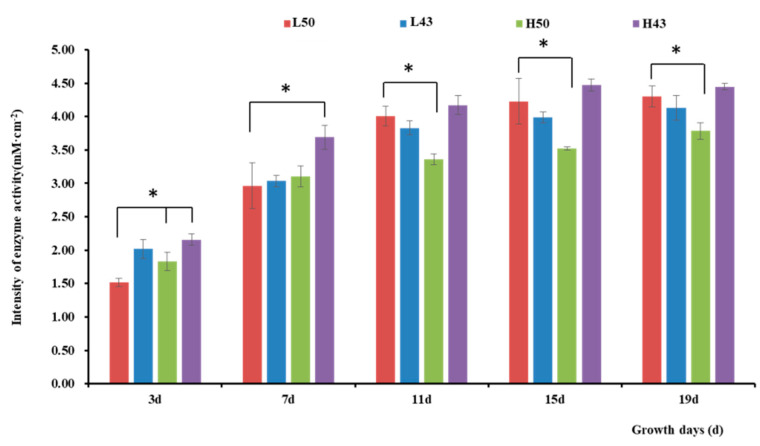
Intensity of β-glucosidase activity in the root tips. * indicates significant at the 0.01 probability level.

The enzyme spectroscopy results of DN50 and Heihe43 showed that the change in enzymatic activity followed a pattern of low enzyme activity in the root base and increasing enzyme activity toward the root tip, with the highest enzymatic activity at the root tip forming an enzyme activity hotspot (Figure 1, Figure 2 and Figure 3; 50 indicated DN50 and 43 indicated Heihe43, respectively; L indicated the cool-temperature environment, and H indicated the warm-temperature environment; * indicates significant at the 0.01 probability level; the same were as follows).

Variety-dependent differences were observed between DN50 and Heihe43. DN50 under a cool-temperature environment had the greatest increase in enzyme activity at the root base and tip from Day 3 to Day 19, with increases of 2.76 mM·cm^−2^ and 2.79 mM·cm^−2^, respectively. When measured at 3 d and 7 d under a cool-temperature environment, the enzymatic activity observed at the root base and tip of DN50 was lower than that under a warm-temperature environment, and the enzymatic activities at both segments were enhanced more under cool-temperature environments, including 11 d, 15 d, and 19 d. Heihe43 exhibited higher root system enzymatic activity under warm-temperature environments.

#### 2.1.3. The Change in Enzyme Activity Strength along the Lateral Root System of Soybeans

Due to the large number of lateral roots showing a great impact on plant growth and development, five complete lateral roots were taken and observed in the current study. The lateral roots were also divided into upper segments (near the root base) and lower segments (near the root tip). The data within each segment were averaged, and single lateral root data were analyzed to obtain the longitudinal change in enzymatic activity along the root system, similar to the taproot system.

**Figure 4:**
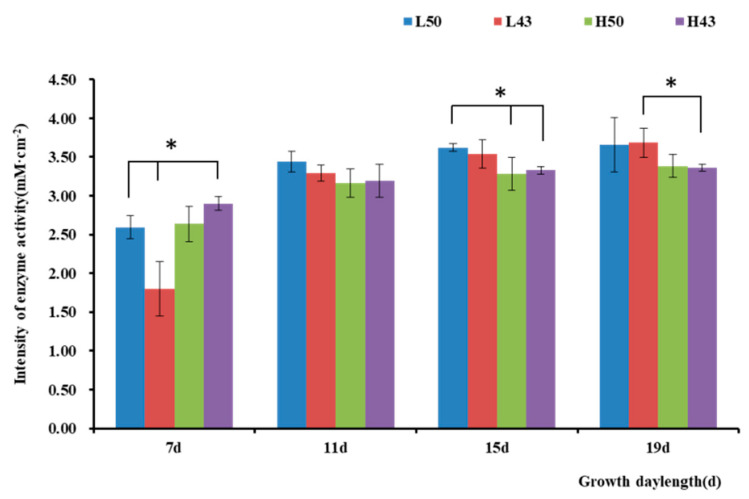
Intensity of β-glucosidase activity along the lateral roots. * indicates significant at the 0.01 probability level.

**Figure 5:**
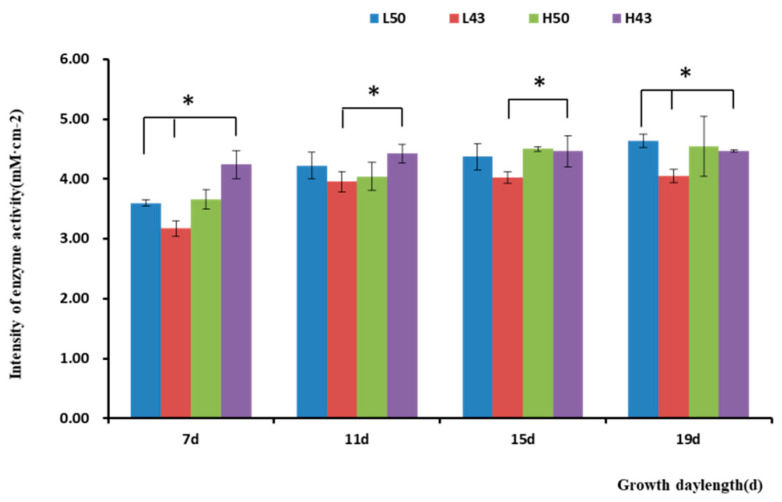
Intensity of β-glucosidase activity in the root tips of the lateral roots. * indicates significant at the 0.01 probability level.

The enzyme activity observed in the lateral roots was similar to that in the taproots (Figure 4 and Figure 5), with the trend of increasing enzymatic activity from the root base to the root tip, forming an enzyme activity hotspot at the root tip. The largest change in β-glucosidase activity in the root base of the Heihe43 variety grown under low-temperature conditions was 1.88 mM·cm^−2^, and the largest change in β-glucosidase activity in the root tip of the DN50 variety grown under cool temperature conditions was 1.04 mM·cm^−2^. In the root base, the enzymatic activities of the DN50 and Heihe43 varieties grown in a cool-temperature environment were lower than those under a warm-temperature environment. In the root tip, the enzymatic activities of the DN50 and Heihe43 varieties grown under high-temperature treatment were generally higher than those of the control group grown under low-temperature treatment. All these results indicated that a warm-temperature environment was more conducive to the secretion of β-glucosidase at the root tip. In the root base, root growth during the early stages was more conducive to the secretion of β-glucosidase, while during the later growth stage, a cool-temperature environment was more conducive to enzyme secretion.

**Figure 6:**
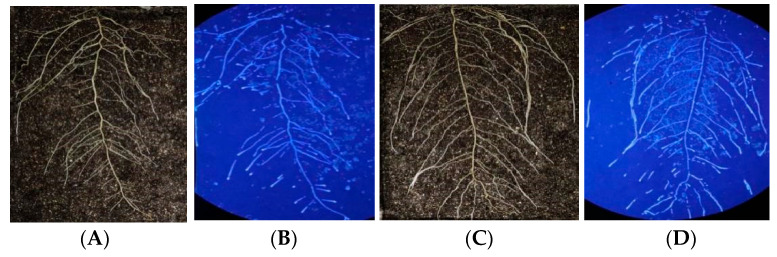
Root structure and in situ β-glucosidase profile of DN50 and Heihe43 grown at high and low temperatures. The left side of the figure depicts the root system. On the right is the RGB image determined via enzyme spectrometry. (**A**) DN50 at a low temperature; (**B**) Heihe43 at a low temperature; (**C**) DN50 at a high temperature; and (**D**) Heihe43 under a high temperature.

Comparing β-glucosidase activity under cool and warm-temperature environments (Figure 6), it was observed that the enzyme activity varied depending on the root segment. The root base diameter was the largest, while the enzyme activity was the weakest, which should be due to the high lignification level, resulting in weak metabolic activity and fewer enzymes being secreted into the rhizosphere. With the longitudinal extension of the taproot system, enzyme activity gradually increased. In the root tip, the taproot diameter was the smallest, while the meristem region exhibited the most vigorous metabolic activity, forming an enzyme activity hotspot. The lateral root was basically consistent with the primary root, suggesting that the enzymatic activities were consistent with the root cell activities.

#### 2.1.4. Exploration of Enzyme Kinetics in Soybean Rhizosphere and Non-Rhizosphere Soil

Generally, the activity of soil enzymes increased with increasing temperature within a certain temperature range and then decreased after reaching the optimum temperature, as determined by analyzing the β-glucosidase activity in the rhizosphere soil of DN50 and Heihe43 measured at various time points (Table 1). When the substrate concentration was within 0–20 μmol·L^−1^, enzyme activity increased with increasing temperature. However, when the substrate concentration exceeded 40 μmol·L^−1^, enzyme activity decreased with increasing temperature. We speculated that within 20–40 μmol·L^−1^, β-glucosidase completely reacted with the substrate. In addition, enzyme activity increased with increasing substrate concentration.

**Figure 7:**
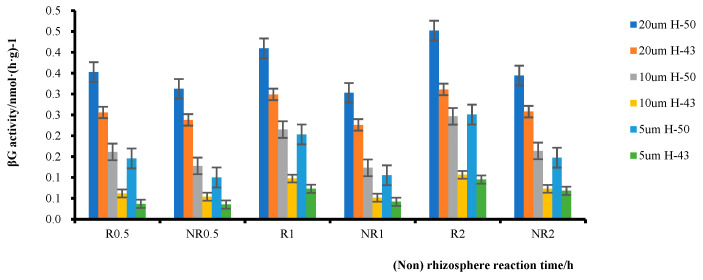
Enzymatic activities in the rhizosphere soil of two soybean varieties at different times and temperatures.

The substrate concentration range of 0–20 μmol·L^−1^ was selected as a reasonable range for further investigation. The β-glucosidase activity in rhizosphere and non-rhizosphere soils within this substrate concentration range was analyzed (Figure 7). The enzyme activity of the soybean rhizosphere soil was significantly higher than that of the non-rhizosphere soil. At a substrate concentration of 5 μmol·L^−1^, Heihe43 had a 4.23% higher Rβ/NRβ ratio than DN50 at 0.5 h, a 74.29% higher ratio at 1 h, and a 39.27% higher ratio at 2 h. DN50 had a 45.36% higher Rβ/NRβ ratio than Heihe43 at 0.5 h, a 92.79% higher ratio at 1 h, and a 69.80% higher ratio at 2 h. The Rβ/NRβ ratio of both varieties exhibited similar trends under other substrate concentrations, i.e., the difference between Rβ and FRβ at 1 h was significantly higher than that at 0.5 h and 2 h; this indicated that there was a peak in the reaction between 0.5 h and 2 h, after which the rate of increase in rhizosphere enzyme activity slowed. In addition, a comparison between DN50 and Heihe43 revealed that at a substrate concentration of 5 μmol·L^−1^, the Rβ/FRβ ratio of DN50 was 45.36% higher than that of Heihe43 at 0.5 h and that of Heihe43 was 4.22% higher. At a substrate concentration of 10 μmol L^−1^, the Rβ/NRβ ratio of DN50 was 26.29% higher than that of Heihe43, showing a 13.97% increase compared to Heihe43. At a substrate concentration of 20 μmol·L^−1^, the Rβ/NRβ ratio of DN50 was 12.90% higher than that of Heihe43 and that of Heihe43 was 7.48% higher. Similar trends were observed at 1 h and 2 h, indicating that DN50 was more sensitive than Heihe43 in the rhizosphere.

### 2.2. Rhizosphere Soil Microbial Diversity

#### 2.2.1. Depth Assessment of Soil Sample Sequencing

Through Illumina MiSeq high-throughput sequencing, a total of 1,556,881 effective sequences were obtained from soil samples, of which 1,551,330 high-quality sequences were obtained after filtering and removing chimeras, and the length of the high-quality sequences was concentrated between 400 bp and 500 bp, divided by 99.64% similarity (Table 2).

**Figure 8:**
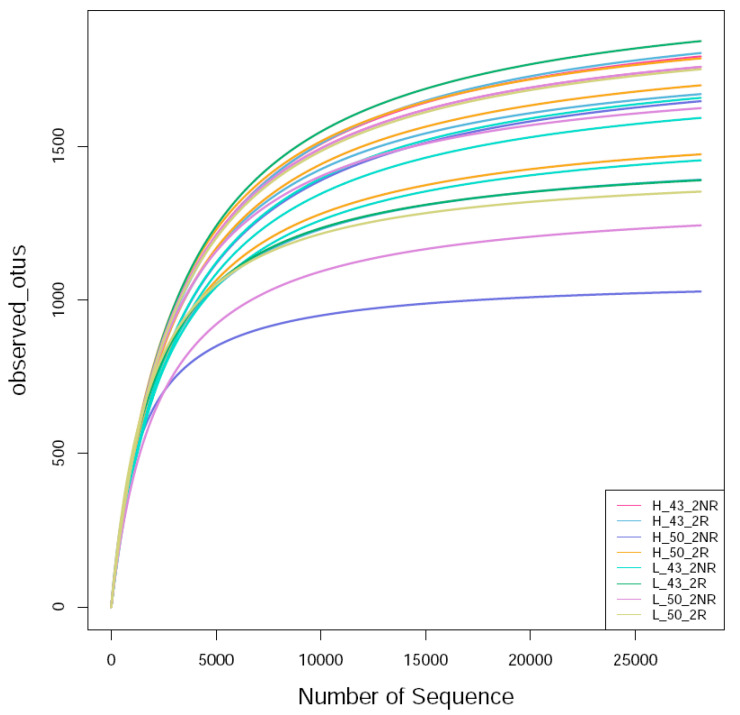
Bacterial rarefaction curve of the soil sample. H_43_2NR indicated the soil sample collected from the non-rhizosphere of Heihe43 in a warm-temperature environment, and H_50_2NR indicated the soil sample collected from the soybean variety DN50; H_43_2R indicated the soil sample collected from the rhizosphere of Heihe43 in a warm-temperature environment, and H_50_2R indicated the soil sample collected from the soybean variety DN50; L_43_2NR indicated the soil sample collected from the non-rhizosphere of Heihe43 in a cool-temperature environment, and L_50_2NR indicated the soil sample collected from the soybean variety DN50; L_43_2R indicated the soil sample collected from the rhizosphere of Heihe43 in a cool-temperature environment, and L_50_2R indicated the soil sample collected from the soybean variety DN50. The same were as follows in Figure 9 and Figure 10.

The raw label data (raw labels) of each sample were filtered and clustered as operational taxonomic units (OTUs). Then, Usearch was employed to cluster the sequences at 99.64% similarity, and after filtering out the chimeric sequences, the OTUs used for species classification were obtained. Each OTU was considered to represent a species. The sequences obtained via sequencing were randomly sampled; eight bacterial samples were sequenced, and a total of 1,963,080 raw rags were generated after double-ended read splicing. A total of 1,556,881 clean tags were obtained after sequence optimization, and a total of 35,337 OTUs were obtained after clustering. Referring to the literature [36], the sequencing sequences were randomly sampled. The number of sequences drawn and the number of OTUs they could represent were constructed to construct a curve, and the OTUs were clustered at the 97% similarity level, and the dilution curves of each sample were made (Figure 8). It can be seen that the bacterial curve gradually flattened, indicating that the number of sequencing events was reasonable, and more sequencing data will only produce a small number of new OTUs.

#### 2.2.2. Changes in Soil Microbial Community Richness and Diversity in the Soybean Rhizosphere

There were differences in the number of OTUs, Chao1 index, Shannon index, and Pixel uniformity index of soybean rhizosphere soil and soybean non-rhizosphere soil samples (Table 2), and the diversity index of the rhizosphere soil microbial community was higher than that of the non-rhizosphere soil microbial community. The species richness of soybean rhizosphere soil was higher than that of non-rhizosphere soil. The difference in the richness of the two soil microbial communities was not very large, and the biodiversity in the soil microbial community of the rhizosphere was only slightly higher than that in non-rhizosphere soil. The uniformity of the two soil samples was also different, and the uniformity in the soil microbial community of the rhizosphere was also higher than that of non-rhizosphere soil. The species diversity of soil bacterial communities varied significantly between rhizosphere soil and non-rhizosphere soil.

#### 2.2.3. Composition of the Soybean Rhizosphere Soil Microbial Community

**Figure 9:**
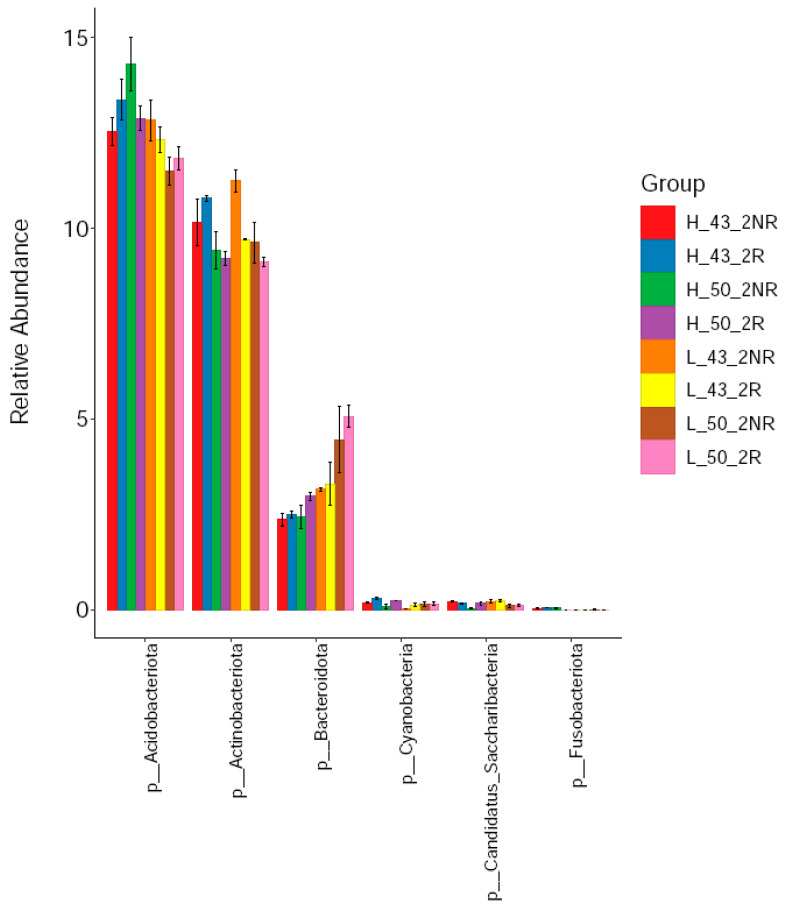
Soil bacterial communities at the phylum level.

We compared the relative abundance of bacteria in rhizospheric and non-rhizospheric soils under high- and low-temperature conditions using the DN50 and Heihe43 varieties. From the phylum classification level, six phyla of bacteria were detected in the rhizosphere soil, including *Acidobacter*, *Actinomycetes*, *Bacteroides*, *Candidatus_Saccharibacteria*, *Cyanobacteria*, and *Fusobacteria*. Among them, the *Acidobacterium phylum*, *Actinomycete phylum*, and *Bacteroides* were the dominant strains in rhizospheric soils (Figure 9), and the relative abundance of the *Acidobacterium phylum* in the rhizosphere soil samples was 12.69%, the relative abundance of *Actinomycetes* was 9.90%, and the relative abundance of *Bacteroides* was 3.28%. Among the dominant species, the relative abundance of *Acidobacterium* and *Actinomycetes* in the rhizosphere soil was higher than that in the non-rhizosphere soil, and the relative abundance of *Bacteroides* in the rhizosphere soil was lower than that in the non-rhizosphere soil. The composition of bacterial communities in rhizosphere and non-rhizosphere soils was significantly different.

**Figure 10:**
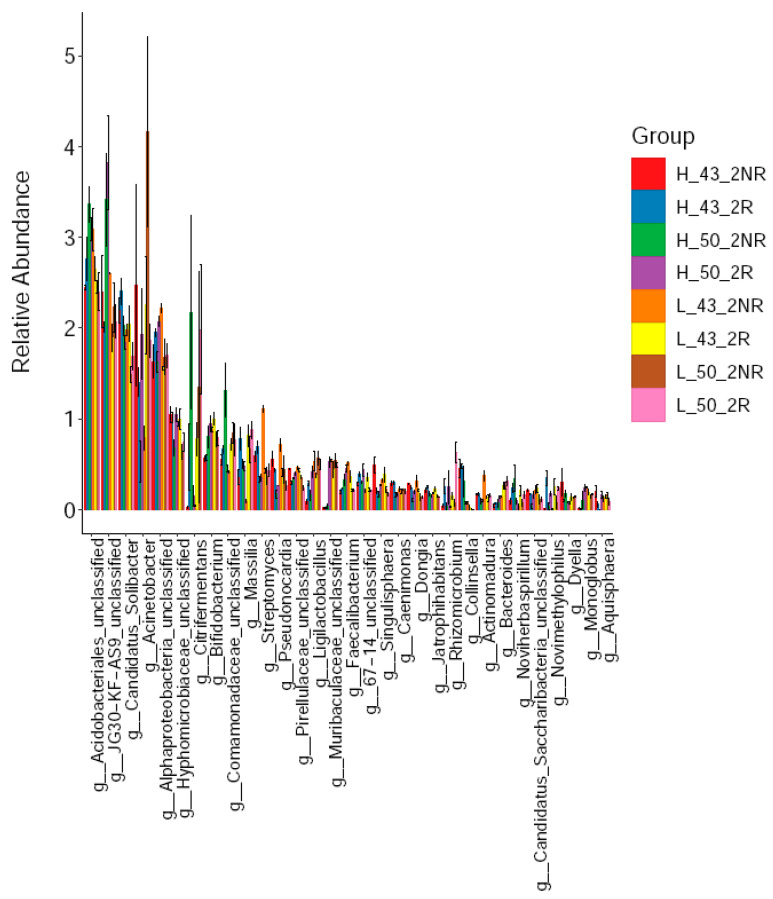
Soil bacterial communities at the genus level.

Subsequently, we further compared the dominant bacterial taxa in the rhizospheric soils of the two soybean varieties under high and low temperature conditions. We found that at the genus level, the dominant taxa of bacteria in different soil samples were significantly different (Figure 10). Among them, the top 15 dominant taxa of rhizospheric soils were *Acidobacteriales* (2.72%), *JG30-KF-AS9* (2.45%), *Candidatus_Solibacter* (2.00%), *Acinetobacter* (1.86%), *Alphaproteobacteria* (1.82%), *g_Hyphomicrobiaceae_ unclassified* (0.95%), *g_Citrifermentans* (0.87%), *g_Bifidobacterium* (0.83%), *g_Massilia* (0.74%), *g_Comamonadaceae_unclassified* (0.65%), g_Streptomyces (0.48%), g_Ligilactobacillu (0.40%), and *g_Muribaculaceae_unclassified* (0.37%), *g_67-14_unclassified* (0.35%), *g_Pseudonocardia* (0.34%), *g_Pirellulaceae_unclassified* (0.34%); non-rhizosphere soil dominant taxa were Acidobacteriales (2.84%), JG30-KF-AS9 (2.66%), *Acinetobacter* (1.99%), *Candidatus_Solibacter* (1.92%), *Alphaproteobacteria* (1.79%), *g_Citrifermentans* (0.90%), *g_Hyphomicrobiaceae_unclassified* (0.83%), *g_Comamonadaceae_unclassified* (0.78%), *g_Bifidobacterium* (0.76%), *g_Streptomyces* (0.60%), *g_Pseudonocardia* (0.47%), *g_Massilia* (0.41%), *g_Pirellulaceae_unclassified* (0.40%), *g_Ligilactobacillus* (0.34%), and *g_Faecalibacterium* (0.31%).

Among these advantageous groups, *Muribaculacea* was present in the rhizospheric soil but not in the non-rhizospheric soil. *Muribaculacea* belonged to the order *Bacteroidales* in the phylum *Bacteroidetes*. The relative abundance of JG30-KF-AS9 in non-rhizospheric soil was 2.66%, while in rhizospheric soil, it was 2.45%. Regarding the relationship between JG30-KF-AS9 and β-glucosidase, it has been proven that β-glucosidase is a core enzyme in soil carbon cycling [37] and an important indicator of general microbial activity [38]. In terms of carbon cycling, the importance of soil microorganisms in many ecosystems depends on the decomposition of cellulose in plant cell walls [39], which is one of the most common organic compounds in the biosphere [40]. β-glucosidase plays a role in the final stage of cellulose degradation in soil, providing an important energy source for microorganisms. Multiple microorganisms are involved in β-glucosidase activity in soil. Achal conducted a multivariate CCA analysis to associate soil health indicators with a large number of soil microorganisms and crop rotation treatments. The results showed that the yield of the high-yield rotation group (CCC and SCC) was related to protein and POX-C, while the low-yield rotation group (CSC and SCS) was related to β-glucosidase. The bacterial group JG30-KF-AS9 enriched in the CCC plot was negatively correlated with β-glucosidase [41]. Therefore, the significantly higher relative abundance of JG30-KF-AS9 in non-rhizosphere soil than in rhizosphere soil indicated that the β-glucosidase activity of rhizospheric soil was significantly higher than that in non-rhizospheric soil, confirming the research significance of β-glucosidase.

### 2.3. Soil Total Organic Carbon Determination

The total organic carbon in the rhizosphere and non-rhizosphere soils of the soybean plant was measured using a total organic carbon analyzer following seed emergence for 19 days (Table 3). The total organic carbon content of rhizospheric soil was 18.02‰ in a warm-temperature environment and 17.45‰ in a cool-temperature environment. The total organic carbon content of rhizospheric soil under a warm-temperature environment was higher than that under a cool-temperature environment. When grown under a warm-temperature environment for 19 days, the organic carbon content of non-rhizospheric soil was 16.86‰, and that in a cool-temperature environment of non-rhizospheric soil was 16.22‰, suggesting that a warm-temperature environment also benefits total organic carbon accumulation. Moreover, the organic carbon content of the rhizospheric soil was higher than that of the non-rhizospheric soil under a warm-temperature environment.

The organic carbon in soybean rhizosphere soil mainly comes from soybean root exudates and soil microorganisms, while the organic carbon in non-rhizosphere soil comes from soil microorganisms. Soil hydrolytic enzymes such as β-glucosidase participate in the decomposition of various glucosides and organic carbon. β-glucosidase was positively correlated with soil organic carbon content [42]. Therefore, it could be inferred that compared with a cool-temperature environment, soybean roots under a warm-temperature environment were more conducive to the secretion of β-glucosidase and exhibited stronger enzymatic activity (Table 4).

## 3. Discussions

### 3.1. The Important Role of Roots in Plant Growth

Plant roots not only anchor deep into the soil to support the vertical growth of plants but also extend radially into the surrounding soil to increase the root surface area and absorb more water and nutrients [43], including organic carbon. Many researchers have correlated morphological changes with root traits, including root length, root weight, and root surface area, and yield accumulation to provide guidance for practical production [44,45]. Researchers have also summarized the evolution of soybean roots in different years and found that soybean varieties tend to develop towards increasing root weight, root surface area, and lateral root length, resulting in a higher absorption ability for water and nutrients, which was positively correlated with yield [46]. This result indirectly illustrates that the growth status of roots have a direct impact on crop yield formation. In the current study, two soybean varieties, DN50 and Heihe43, with varying dry root weights were observed by uprooting and photographing them. The root systems of Heihe43 soybeans grew more vigorously than those of DN50, with more lateral roots and nodules. The stems of Heihe43 soybeans were also thicker and taller than those of DN50 soybeans, suggesting a relation between the root and the lodging resistance of soybean plants.

### 3.2. Effect of Temperature on Rhizosphere Enzymatic Activity

The β-glucosidase activity of the taproot and lateral root of soybeans differed between the root base and the root tip. Soil enzymatic activity fluctuates with root development or non-biological factors, including temperature, pH, moisture, and soil depth [47]. The β-glucosidase activity of the taproot was higher than that of the lateral roots as time passed at the root base, but this trend reversed at the root tip, where the β-glucosidase activity of the taproot was lower than that of the lateral roots. The fluctuation of β-glucosidase activity was consistent with its biological role, including microorganism abundance and diversity, resulting in a change in organic matter in the rhizosphere. Temperature had a direct effect on soil enzymatic activity and was an important environmental factor affecting soil enzymatic activity. Within a certain temperature range, soil enzymatic activity increased with increasing temperature and then decreased after reaching the optimum temperature. The warm-temperature environment affected soil enzyme activity by influencing enzyme kinetics and indirectly affected enzyme activity by affecting soil microbial biomass and community structure. In addition, increasing soil temperature affected various chemical reactions and ion compositions of the soil solution, increasing soil nutrient availability and microbial biomass and thereby increasing soil enzyme activity, suggesting that temperature rises play an important role on the biological activity in the rhizosphere and facilitate the accumulation of organic matter.

In warm-temperature environments, microbial communities and plant physiological activities gradually change. Therefore, different durations of warm temperatures led to different results. For example, short-term warming led to a decrease in β-glucosidase activity [48]. In addition, several studies have shown that warming can indirectly affect soil enzyme activity by affecting biomass allocation and nutrient utilization, causing changes in root exudates [49]. In the current study, β-glucosidase activity showed a variety-dependent and development-stage difference. When the roots grew for 7 days, the β-glucosidase activity of rhizosphere soil was lower than that of non-rhizosphere soil, and the β-glucosidase activity of DN50 was higher in a cool-temperature environment, while the β-glucosidase activity of Heihe43 was higher in soils under a warm-temperature environment. When the roots grew for 21 days, β-glucosidase activity in rhizosphere soil was higher than that in non-rhizosphere soil, and both DN50 and Heihe43 exhibited higher β-glucosidase activities in cool-temperature environments, suggesting that the difference in enzyme activity between rhizosphere and non-rhizosphere soils was related to root exudates and microbial distribution. The differences in temperature and root exudates affected the distribution of microbial populations, resulting in differences in soil enzymatic activity at different developmental periods.

### 3.3. Soil Microbial Community Structure

The soil microbial community is the foundation of soil ecological functions, affecting soil nutrient cycling and regulating and indicating soil functions through participation in organic matter decomposition and mineralization processes [50]. There are a large number of microorganisms in the soil, with bacteria being the most numerous in terms of species and quantity. Bacteria play a crucial role in the formation and decomposition of organic matter, the circulation of soil nutrients, the maintenance and improvement of soil fertility, the growth and development of plants, the prevention and control of pests and diseases, and the improvement of the ecological environment [51]. The sequencing results showed that *Acidobacteria*, *Actinomycetes*, and *Planctomycetes* were the dominant bacterial species in soybean rhizospheric soil, with *Acidobacteria* having the highest relative abundance at 12.69%. This was consistent with the results of Peralta et al. [52]. *Acidobacteria* degrade complex lignin [53] and cellulose [54], providing nutrients to the soil, and the decomposition of residual soybean plants by *Acidobacteria* provides sufficient energy for soil microorganisms [55]. In the current study, the number of OTUs, Chao1 index, Shannon index, and Pielou’s evenness index of soybean rhizosphere soil and non-rhizosphere soil samples were compared, and all indices of soybean rhizosphere soil were higher than those of non-rhizosphere soil, indicating that the microbial community diversity in rhizosphere soil was higher and the microbial community was more stable.

The interaction between soil microbial communities and plant health is closely related. Studies have shown that the diversity of immune-type rhizosphere microbial communities is higher, and the microbial interaction network is more complex [56]. The soil microbial community is the core and key to maintaining soil health. Using the soil microbial community to improve soil health is of great significance for sustainable agricultural development and soil ecological environment protection. Enriching specific functional microbial groups through artificial regulation, cultivating specific functional microbial communities, and constructing an immune-type rhizosphere soil microbial ecosystem are new technologies and approaches to reduce the use of chemical pesticides and improve soil health.

### 3.4. Soil Total Organic Carbon

Soil organic matter is the main source of enzyme substrates in soils. Soil organic matter is the storage place of organic carbon, while soil total organic carbon is used as a chemical measure of organic matter [57]. Soil enzymes, as one of the active organic components of soils, participate in the soil organic carbon cycling process in different environments. Soil enzymes may mainly affect the conversion of soil organic carbon through indirect effects, such as increasing the leaching of nutrient elements in soil through enzymatic action and activating or inhibiting soil microbial activity. In this work, the rhizosphere total organic carbon content was higher than that of the non-rhizosphere total organic carbon content under warm-temperature environments. Based on soil enzyme kinetics, it was concluded that rhizosphere soil enzyme activity was higher than that of non-rhizosphere soil. Combining the results of the two experiments, it was easy to observe that soil enzyme activity was positively correlated with total organic carbon content [54]. Various soil enzymes actively participate in the conversion of soil organic carbon, which plays an important role in improving soil fertility. The organic carbon content was also the basis of soil enzyme activity, which had an undeniable influence on soil enzyme activity. The carbon, nitrogen, phosphorus, and other elements that are contained in soil microorganisms themselves are regarded as reserves of soil nutrients and participate in material and energy exchange in soils [58]. There was a significant correlation between the accumulation mineralization of surface soil organic carbon and the activity of β-glucosidase, and enzyme activity directly affected the microbial degradation of organic matter [59]. Soil microbial communities play a key role in regulating carbon fixation and decomposition. Increasing the abundance and diversity of soil bacteria while reducing the number of fungi, inhibiting soil microbial respiration, and tending to use easily decomposable organic matter could slow soil carbon decomposition and promote the accumulation of soil organic carbon [60]. In addition, β-glucosidase is an important indicator for evaluating soil fertility, and the higher the organic matter content, the stronger its enzyme activity [61].

In recent years, changes in root microbial communities have received substantial attention as drivers of plant–soil feedback [62,63]. Various plant health benefits have been associated with the rhizosphere microbiome [10], and plant–soil feedback represents a promising way to harness these positive effects, including growth promotion and insect resistance in agricultural settings [64,65]. Compared with non-rhizosphere soil, the rhizosphere area was more nutrient rich, and plant cell debris and root exudates provided abundant carbon sources and other nutrients for rhizospheric microorganisms. Rhizosphere soil microorganisms influence plant growth by affect the decomposition of soil organic matter [66]. In most soils, microbial populations are limited by the lack of carbon sources, thereby affecting the decomposition of organic matter [67]. Roots change the activity of rhizosphere soil microorganisms by depositing organic carbon components, competing with nutrients, and changing rhizosphere physicochemical properties, which, in turn, affect the degradation of soil organic matter. The decomposition of organic matter provides nutrients for plant growth, which secrete carbon and energy for rhizosphere microorganisms. Following this, rhizosphere microorganisms accelerate the degradation of soil organic matter and return mineral nutrients to plants.

The two soybean varieties, Heihe43 and DN50, showed similar changes in various rhizosphere indicators, but Heihe43 was more resistant to lodging and had a higher yield; this was because Heihe43 had a well-developed root system, a larger surface area, higher dry weight, and greater biomass than DN50. Therefore, soybean lines with well-developed root systems should be considered during high-yield soybean variety breeding.

### 3.5. Summary of the Impact of Warm Environmental Temperature on the Rhizosphere in Soybean Seedling Roots

**Figure 11:**
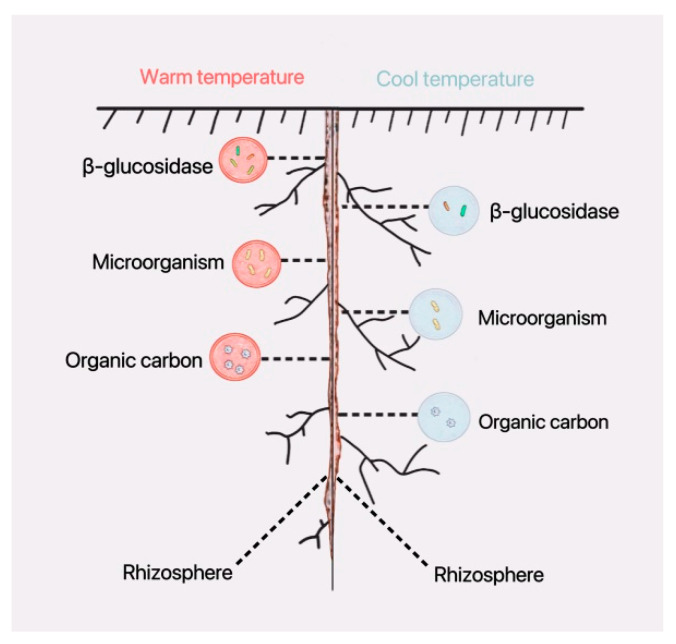
A warm environmental temperature strengthens the biological activities on the rhizosphere in soybean seedling roots. The pink circle indicates stronger biological activities in a warm-temperature environment, and the blue circle indicates weaker biological activities in cool-temperature environment.

Soil enzymes are involved in the cycling of organic carbon in the rhizosphere, and the higher the organic carbon content, the higher the soil enzyme activity. Soil enzyme activity is positively correlated with organic carbon content. Increased microbial (bacterial) diversity promotes organic carbon accumulation. Warm environmental temperatures directly affect the composition and structure of microbial communities, leading to increased microbial diversity; this, in turn, indirectly enhances soil enzyme activity and organic carbon content (Figure 11).

## 4. Materials and Methods

### 4.1. Plant Materials

Two soybean varieties, DN50 with low root dry weight and Heihe43 with high root dry weight, were chosen to observe the ambient temperature increase on various spatiotemporal configurations of soybean roots.

### 4.2. Method for Root Growth in the Root Cassette

The millisoil was air-dried and ground to a powder of 200 mesh and mixed with 13% zeolite (weight) as an equal weight of rock, and other impurities were disposed. The soil–zeolite mixture was filled in a root box (23 cm × 17 cm × 3 cm) to 2 cm away from the upper end of each root box. The open side was closed, and the root box was slowly raised and kept 60° from horizontal. The root box was strictly sealed on both sides. The root box was allowed to absorb water until the relative moisture content reached 60% to ensure seed germination. Each treatment was replicated at least three times.

### 4.3. Soybean Seeding Growing Conditions

Healthy soybean seeds were selected, and the seed hilum was faced downwards to facilitate seed germination. The root box was placed in an incubator with a daytime temperature of 23 °C (cool temperature) and 28 °C (warm temperature) and a nighttime temperature of 18 °C to simulate the temperature environment of the thriving growth stage (R1 or so) of soybeans. The daytime was 14 h in duration, and the nighttime was 10 h duration. The photosynthetic effective radiation intensity was 500 μmol m^−2^ s^−1^. The roots tended to grow towards the surface soil due to the gravity effect. Plant growth was monitored and observed during the cultivation period.

### 4.4. Soil Enzyme Activity In Situ Measurement

#### 4.4.1. Enzyme Spectrum Analysis and UV Imaging

The rhizosphere enzyme activity test followed the procedure of Kuzyakov’s Lab [68] with a slight modification. 4-Methylumbelliferyl-β-D-glucoside (MUF-G) was used as the substrate to conduct the in situ enzyme activity of β-glucosidase. The substrate was dissolved in a 12 mmol·L^−1^ MES buffer (MES hemisodium salt). The polyamide filter membrane was soaked in the substrate solution for 20 min. The soaked saturated membrane was then placed on the soil surface and incubated for 90 min. After incubation, the membrane was placed under UV irradiation in a darkroom to observe the fluorescence data (with an excitation wavelength of 355 nm and an emission wavelength of 460 nm).

#### 4.4.2. Processing and Analysis of Enzyme Spectrum Images

Under UV light, the position where the fluorescent substance (MUF) carried in the substrate shows a fluorescent reaction is the area where the substrate is hydrolyzed by a specific enzyme. The strength of the fluorescent reaction is positively proportional to enzyme activity. Image J vl.46 was used to process the enzyme spectrum image and analyze the data.

To calculate enzymatic activity, a standard function curve was established. The operation method was as follows: prepare various concentrations of MUF solutions (0.01, 0.10, 0.20, 0.25, 0.50, 1.00, and 2.00 mmol·L^−1^). The membrane was cut into 1 cm^2^ pieces and immersed in gradient MUF solutions. The saturated membrane was placed under UV light, and the average gray value of the saturated fluorescent area was obtained. The “actual concentration = C (MUF aq)·S^−1^ (saturated fluorescent area) (mM·cm^−2^)” was used as the *X*-axis, and the “actual gray value = average gray value of saturated fluorescent area—background gray value” was used as the *Y*-axis to obtain the standard curve. The standard curve was used to analyze the experimental images and obtain the enzyme activity data, with units of mM·cm^−2^. The standard curve equation was established as follows: $y=\frac{x\;-\;0.1903}{19.523}$, where x is the pixel-level grayscale value of the fluorescence reaction site in the image, and y is the actual quantitative enzyme activity represented by that site.

#### 4.4.3. Extraction of Soil Microbial Genomic DNA

The extraction of DNA was carried out using the PowerSoil DNA isolation kit (MOBIO, Jefferson City, MO, USA). The universal primers 27F (5′-AGAGTTTGATCCTG-GCTCAG-3′) and 1492R (5′-TACTTGTTACGACTT-3′) [69] were used to amplify the soil bacterial 16S rDNA gene. The PCR system was 20 μL in total, including 2 μL of DNA templates (100 ng), 2 μmol·L^−1^ of primers, 2 μL of 10 × AccuPrime PCR Buffer II, 0.15 μL of Accuprime Taq Hifi (Thermo Fisher, Waltham, MA, USA), and 13.85 μL of RNAse/DNAse-free water. The PCR program was as follows: 95 °C for 2 min; 95 °C for 20 s, 50 °C for 30 s, and 72 °C for 5 min (30 cycles), and 72 °C for 10 min. Three replicates were performed for each sample, and the PCR products of the same sample were mixed and purified via gel excision after electrophoresis.

#### 4.4.4. Determination of Organic Carbon

Each soil sample was divided into two subsamples: one was used to test the percentage of total carbon (TC) in the bulk sample after processing, and the others were used to test the percentage of organic carbon (TOC) in the carbonate-free residue after processing. Carbon has two forms: organic carbon (OC) and inorganic carbon (IC), and TIC is the total inorganic carbon amount. TOC = TC − TIC. The specific steps were as follows: 1 g of the sample was weighed and dried at 50 °C for 24 h; the sample was ground to a powder of 200 mesh, and 1 mol/L hydrochloric acid was added to remove the carbonate and stirred with a magnetic stirrer for 24 h, which was repeated 4–5 times. The sample was washed with deionized water and centrifuged, and the supernatant was discarded. The pH was turned to 7.0. After drying for 24 h, the sample powder was placed into a dryer after grinding and weighed after 12 h. Then, a total organic carbon analyzer was used to test it using the combustion method.

#### 4.4.5. Enzyme Kinetics Determination

Rhizosphere soil and non-rhizosphere soil were collected after seed germination for 19 days. First, 0.1 g of rhizosphere and non-rhizosphere soils were carefully collected from each root box with a needle. Then, a suspension of 0.1 g of soil was prepared in 10 mL of deionized water using the low-energy ultrasound treatment (40 J/S output energy) for 2 min. Then, 50 μL of the soil suspension, 100 μL of a series of substrate component concentrations (0 μM, 5 μM, 10 μM, 20 μM, 40 μM, 80 μM, 100 μM, and 150 μM), and 50 μL of buffer (MES or TRIZMA) were added to a 96-well microplate. The fluorescence data were recorded using a multifunctional microplate reader (Spark, Tecan, Switzerland) at an excitation wavelength of 355 nm and an emission wavelength of 460 nm, with a slit width of 25 nm. All enzyme activities were measured after 30 min, 1 h, and 2 h after adding the soil solution, buffer, and substrate solution. The blank was determined using the enzyme kinetics of root box soil without plants. Enzyme activity was expressed as MUF (nmol/g dry soil) released per hour per gram of dry soil. Each enzyme at each substrate concentration was determined four times.

#### 4.4.6. Data Processing and Analysis

Enzyme spectrum images were processed using Microsoft Excel 2021, Photoshop 6.0, and Image J vl.46.

The raw data obtained from sequencing were spliced using FLASH [70] to obtain the raw tag data (raw tags) of each sample. The spliced raw tags were then subjected to strict filtering [71] to obtain high-quality clean tag data (clean tags). The quality-controlled tags were then processed using the tag quality control pipeline of QIIME [72], and the chimeric sequences were removed. The tag sequences were aligned with the UNITE database using the UCHIME algorithm [73] to detect chimeric sequences, which were then removed [74] to obtain the final effective data. To facilitate downstream species diversity analysis, the tags were clustered into operational taxonomic units (OTUs). First, singletons (sequences with only one read) in the spliced tags were filtered out, as singletons may be caused by sequencing errors. Then, USEARCH v5.2.32 was used to cluster the sequences at 99.64% similarity, and after filtering out the chimeric sequences, the OTUs used for species classification were obtained. Each OTU was considered to represent a species.

## 5. Conclusions

The in situ results of root enzyme activity revealed that soybean roots secrete β-glucosidase, and enzyme spectrum imaging demonstrated different enzymatic activities in different temperature environments. The soil enzyme kinetics results showed that soil enzyme activity increased with increasing temperature, and soybean rhizosphere soil enzyme activity was higher than that of non-rhizosphere soil. Rhizosphere soil and non-rhizosphere soil showed that the dominant bacterial phylum in soybean rhizosphere soil was *Acidobacteria*, and the dominant bacterial genus was JG30-KF-AS9. Compared with non-rhizosphere soil, rhizosphere soil was more nutrient rich, and root secretions provided abundant carbon sources and other nutrients for soil microorganisms in the rhizosphere. Rhizosphere microorganisms affect plant growth by influencing the decomposition of soil organic carbon. The organic carbon content of rhizosphere soil was higher than that of non-rhizosphere soil under high temperatures. The current study provides a theoretical basis for breeding high-yield soybean varieties in the root rhizosphere in the context of global warming.

## Figures and Tables

**Table 1:** Enzyme activity in the rhizosphere soil of two soybean varieties at different times and temperatures (nmol·(h·g)^−1^).

Time	0.5 h				1 h				2 h			
Concentration Variety	L-50	H-50	L-43	H-43	L-50	H-50	L-43	H-43	L-50	H-50	L-43	H-43
0 (μmol·L^−1^)	0	0	0	0	0	0	0	0	0	0	0	0
5 (μmol·L^−1^)	0.0536	0.1458	0.0661	0.0355	0.0933	0.2032	0.1009	0.0732	0.1150	0.2466	0.1184	0.0954
10 (μmol·L^−1^)	0.0855	0.1614	0.0852	0.0620	0.1275	0.2150	0.1132	0.0977	0.1427	0.2508	0.1244	0.1067
20 (μmol·L^−1^)	0.3254	0.3528	0.2840	0.2559	0.3819	0.4096	0.3225	0.2992	0.3988	0.4520	0.3236	0.3112
40 (μmol·L^−1^)	0.8950	0.7780	0.7542	0.6848	0.9721	0.8418	0.8130	0.7415	0.9642	0.8574	0.8309	0.7336
80 (μmol·L^−1^)	2.6377	2.1136	2.1717	2.0530	2.7469	2.1935	2.2540	2.1401	2.7158	2.1779	2.2005	2.0869
100 (μmol·L^−1^)	3.6003	2.8296	2.9068	2.7595	3.7167	2.9133	2.9383	2.8231	3.6883	2.8542	2.8269	2.7851
150 (μmol·L^−1^)	9.6033	7.3499	7.7105	7.3137	9.7147	7.4045	7.7377	7.3928	9.5107	7.2302	7.6084	7.1510

**Table 2:** The length distribution of the sample sequence.

Length (bp)	Count	Percentage (%)
<200	55	0.0035
200–300	4347	0.2792
300–400	1149	0.0738
400–500	1,551,330	99.6435
≥500	0	0

**Table 3:** OTU abundance and diversity of bacteria in the soil sample.

Soil Sample	No. of OTUs	Chao1	Shannon Index	Pielous Index
Rhizosphere soil	1556.17	1612.94	9.3825 (0.25)	0.89
Non-rhizosphere soil	1338.58	1444	9.0875 (0.45)	0.88

**Table 4:** The organic carbon content of rhizosphere and non-rhizosphere soils in environments with varying temperatures.

	Rhizosphere (‰)	Non-Rhizosphere (‰)
Cool-temperature environment	18.02 ± 0.47	16.22 ± 0.28
Warm-temperature environment	17.45 ± 0.04	16.86 ± 0.29

## Data Availability

Data are contained within the article.
